# Supplementary material for: Space–Time Conditional Autoregressive Modeling to Estimate Neighborhood-Level Risks for Dengue Fever in Cali, Colombia
Source: Am J Trop Med Hyg. 2020 Aug 31;103(5):2040–53. doi: 10.4269/ajtmh.20-0080 (PMC7646775; doi:10.4269/ajtmh.20-0080)
Supplement: Supplementary file 1 [file tpmd200080.SD1.pdf]

Poisson GLM Results

Table A provides the results for the two DENF Poisson GLMs – Model 1: no lags and Model 2: lagged weather variables. All fourteen independent variables in the no lag and lagged models are statistically significant (*p* < 0.001). Furthermore, there was statistically significant evidence of unexplained spatial and temporal autocorrelation in the residuals after conducting a Moran's I test for each week but excluded for brevity. The residual spatial and temporal autocorrelation indicates that it is therefore appropriate to utilize a ST-CAR model in this study.

Table A: DENF GLM Results – Model 1 - No lags; Model 2 - Lagged weather variables

	Model 1				Model 2			
	Coefficient	RR**	Std. Error	*p*	Coefficient	RR**	Std. Error	*p*
Intercept	-14.5553	NA	0.3575	*	-0.7722	NA	0.0471	*
PC1	0.0916	1.0636	0.0097	*	0.0916	1.0636	0.0097	*
PC2	-0.1865	0.8298	0.0161	*	-0.1865	0.8298	0.0161	*
Pnurseries	0.6651	1.9448	0.0340	*	0.6651	1.9448	0.0340	*
Tires	0.1155	1.1225	0.0497	*	0.1155	1.1225	0.0497	*
Popdens	-1.1714	0.3099	0.0596	*	-1.1714	0.3099	0.0596	*
Rivers	-0.0106	0.9894	0.0363	*	-0.0106	0.9894	0.0363	*
Trees	0.3765	1.4571	0.0672	*	0.3765	1.4571	0.0672	*
Tavg(L5)	0.6013	1.8244	0.1460	*	1.5640	4.7779	0.0438	*
DTRMax(L4)	0.9585	2.6077	0.0370	*	0.7221	2.0588	0.0405	*
RelHRng(L3)	0.0077	1.0077	0.0016	*	0.8401	2.3167	0.0312	*
RainT(L3)	0.0063	1.0063	0.0005	*	-1.9794	0.1382	0.0512	*
RainD(L5)	-0.0090	0.9910	0.0054	*	0.0129	1.0130	0.0332	*
CoolD(L2)	0.1465	1.1577	0.0075	*	-0.3471	0.7067	0.0388	*
WarmD(L5)	-0.1838	0.8321	0.0049	*	-0.8104	0.4447	0.0334	*

*Statistically significant at the *p* <0.001 level.
**RR: Relative Risk

PC2, population density, and proximity to rivers all have a negative relationship with DENF, suggesting a decreased risk of DENF transmission. PC1, proximity to plant nurseries, proximity to tire shops, and tree density all have a positive relationship with DENF, suggesting an increased risk of DENF transmission.
